# Naomi Richman, BM, FRCPsych

**DOI:** 10.1192/bjb.2018.74

**Published:** 2019-04

**Authors:** Philip Graham

**Formerly Reader, Institute of Child Health, and Honorary Consultant Child Psychiatrist, Hospital for Sick Children, Great Ormond Street, UK.**


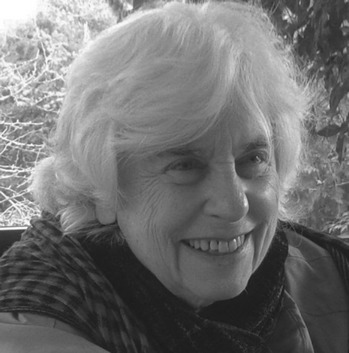


Naomi Richman, who died recently at the age of 84, carried out pioneering studies on the prevalence of emotional and behaviour disorders in pre-school children in the 1970s and 1980s. She was the first author of a monograph published in 1982 *Preschool to School: A Behavioural Study*^1^. A review of this book began by quoting John Ruskin: ‘All books are divisible into two classes: books of the hour and books of all time’. There was no doubt into which category this monograph belonged, nor would her two co-authors – Jim Stevenson and I – have been in any doubt that she was the inspiration behind the study.

The study was carried out while Naomi was based at the Hospital for Sick Children, Great Ormond Street, and then at the Institute of Child Health, London. It involved more than 700 3-year-old children living in Waltham Forest. First, it demonstrated that it was feasible to produce criteria for a ‘case’ in pre-school children. This was achieved by the development of the Behaviour Screening Questionnaire, the first specifically designed for pre-school children. It allowed the calculation of the high prevalence of behaviour disorders in this age group: around 7% for moderate and severe problems. Further, by following children up to 8 years old, she and Jim Stevenson were able to show that, contrary to the then-current popular belief, behaviour problems in pre-school children and the language delay often associated with them were not transient and part of normal development, but often persisted at least well into the primary school years.

This study was the first (although many were later carried out by other researchers) that examined the stresses leading to behaviour problems in very young children. A particular set of problems related to bringing up young children in tower blocks, and much of the concern for family life in such buildings first arose from studies in which Naomi played a leading part. With Jo Douglas, she went on to develop behavioural methods of dealing with sleep problems in early childhood. These were evaluated using controlled designs and found to stand up to rigorous testing. Naomi and Jo published a best-selling Penguin book *My Child Won*'*t Sleep*, which described their techniques, and then ran training workshops for health visitors, enabling them to apply this management approach.

In 1989, Naomi went to work for the Mozambique Ministry of Education, first on sabbatical leave and then full time, on a Swedish Save the Children Fund programme aimed at helping children traumatised by the civil war that was then raging in that country. She developed training programmes for teachers working in areas where large numbers of children had been in direct contact with war and violence. From her experiences in Mozambique, she developed her ideas regarding principles to help children involved in organised violence.

Naomi also worked in Angola (with Pam Zinkin and Nazeen Kanji), Central America, the occupied territories and South East Asia, organising training programmes for children similarly exposed to violence. She was strongly critical of medical approaches to so-called post-traumatic stress disorder. She saw this as an often inappropriately applied diagnosis that implied a psychiatric condition needing specialist therapy, without regard to the frequently ongoing nature of traumas and the ways culture influenced community attitudes towards them. Instead, in her influential articles and manuals, she emphasised the need for traumatised children to be reintegrated into their families and given appropriate education.

In 1996 after returning to the UK, Naomi carried out a comprehensive study of the psychosocial needs of Kurdish, Somali and Vietnamese refugee children and families in Hackney, London. Out of this work, she wrote a guide for professional workers entitled *In the Midst of the Whirlwind: Manual for Helping Refugee Children.*

Naomi was born and brought up in Leeds. Her father, Louis (a shopkeeper), and her mother, Gertrude (Gerti), were second-generation Jewish immigrants from Eastern Europe. She won a place at the local grammar school, Roundhay High School, and from there she went on to read medicine at St. Hilda's College, Oxford. After clinical training at the Middlesex Hospital Medical School, she did house jobs, one of them with the paediatrician Dermod McCarthy who insisted that parents of children in hospital should, contrary to the then-current practice, be allowed to visit at all times of the day.

Her psychiatric training was undertaken at the Maudsley Hospital between 1961 and 1964. After leaving the Maudsley, she spent some years in the USA, some of the time as a research fellow on a course in the epidemiology of mental handicap, which was run by Zena Stein at Columbia University Medical School, New York.

Naomi was active politically throughout her life, both as a left-winger and as a feminist. In 1980 she was one of a small group of women doctors who founded the organisation ‘Women in Medicine’ to combat patriarchal attitudes in the profession and to assist medical women in their careers. She remained highly active in this group until it disbanded in 2002.

She was a woman of wide interests with strong political views, feisty in her support for the underprivileged. A talented artist, she was also a keen and knowledgeable birdwatcher, gardener, theatregoer, swimmer in the Highgate Women's Pond and loyal friend to many. She did not have a family of her own, but developed very close, supportive relationships with her niece, Rachel, and nephew, David. In her later years she suffered from Alzheimer's disease. Not always an easy person, in her last years she became an altogether sunnier, more relaxed personality. Naomi died on 16 June 2018 and is survived by her niece, two nephews and three great-nephews.
